# Fine-Tuning a Small Vision Language Model Using Synthetic Data for Explaining Bacterial Skin Disease Images

**DOI:** 10.3390/diagnostics16040603

**Published:** 2026-02-18

**Authors:** Shiwan Zhang, Abdurrahim Yilmaz, Gulsum Gencoglan, Burak Temelkuran

**Affiliations:** 1 Hamlyn Centre, Department of Surgery and Cancer, Imperial College London, London SW7 2AZ, UK; 2 Division of Systems Medicine, Department of Metabolism, Digestion, and Reproduction, Imperial College London, London SW7 2AZ, UK; 3 Department of Dermatology and Venereology, Medicana Atakoy Hospital, Istanbul 34158, Türkiye

**Keywords:** vision language models, fine-tuning, bacterial skin diseases, dermatology imaging, visual question answering, synthetic data, medical AI

## Abstract

**Background/Objectives:** Vision language models (VLMs) show strong potential for medical image understanding, but their large scale often limits practical deployment. This study investigates whether a compact VLM can be effectively adapted for dermatology, with a focus on explaining bacterial skin disease images. **Methods:** We curate a dataset derived from PMC-OA using the BIOMEDICA dataset and construct PMC-derma-VQA-bacteria by pairing images with inherited figure captions and synthetically generated question–answer (QA) supervision produced by Google’s Gemini model. SmolVLM is fine-tuned under three supervision settings: QA-only, caption-only, and a combined QA+caption strategy. The models are evaluated on a held-out test set for both text-generation quality and diagnostic classification performance. **Results:** QA-only supervision yields the best report-generation performance, while the combined QA+caption setting achieves the highest classification accuracy (70.20%). **Conclusions:** Synthetic QA supervision can meaningfully enhance compact VLMs for medical image understanding and diagnostic support in dermatology.

## 1. Introduction

Skin diseases are a major global health concern, consistently ranking among the leading causes of non-fatal disease burden worldwide, while they also could be life-threatening [1]. Studies show that they affect a large proportion of the global population, with prevalence rates ranging from 30% to 70% across all age groups and regions [2]. However, diagnosing skin diseases remains a significant challenge [3]. This is mainly due to two factors. First, skin diseases are highly diverse, and dermoscopic images are often complex and difficult to interpret [4]. Second, there is a shortage of dermatologists, especially in rural areas, where people have limited access to specialist care, while the cost of consultations continues to rise [5].

In response to the limitations of traditional diagnostic methods, artificial intelligence (AI) has shown great potential in medical image analysis and clinical decision support. Deep learning algorithms have achieved remarkable performance in recent years [3]. In dermatology, AI has been increasingly applied to the analysis of dermoscopic images [6,7], enabling automated lesion detection, segmentation, and classification with accuracy comparable to that of expert dermatologists [8,9].

To further enhance diagnostic capabilities, particularly by integrating visual information with clinical context, VLMs have attracted increasing attention. They are valued for their strong cross-domain generalization and scene understanding capabilities [10]. VLMs have been successfully applied in a wide range of fields, including dermatology, where they support tasks such as skin lesion classification, differential diagnosis generation, and dermatology-specific question answering [11,12,13]. Recent work has also begun to systematically evaluate foundation models in dermatology settings, highlighting performance variability across task hierarchies and model scales [14]. By enhancing image interpretation and expanding remote access to dermatological expertise, VLM-based systems help address both the complexity of diagnostic tasks and the shortage of specialist care [15].

However, most state-of-the-art (SOTA) models are computationally intensive and require time-consuming data processing for widespread deployment. VLMs such as CLIP, Flamingo, and BLIP-2 typically require large-scale multimodal datasets and substantial computational resources during training; for example, thousands of GPU or TPU days are often needed to achieve competitive performance [16,17,18]. Their deployment also remains challenging due to large model sizes and high inference latency, which demand specialized hardware to meet acceptable response times [19]. Such requirements are even harder to satisfy in domains where data and computational resources are inherently scarce. Recent dermatology vision-language datasets provide instruction-style supervision, such as DermaSynth, DermaBench, MM-Skin, and DermaVQA [20,21,22,23]. However, these resources are not built from PMC-OA scientific figures with inherited figure captions and structured metadata, and they do not target domain-focused settings with synthetic QA for small VLM adaptation. Moreover, to the best of our knowledge, there is currently no small VLM specifically for bacterial skin diseases trained with synthetic QA supervision.

To address these computational and data problems, we used a common-purpose VLM, SmolVLM, to fine-tune it on synthetic data. First, we curated a dermatology-specific dataset by extracting bacterial skin disease images from the BIOMEDICA corpus, using disease labels annotated by Gemini to ensure domain relevance. Next, we enriched each image with clinically meaningful synthetic QA pairs generated through Gemini and additional supervision strategies, which served as fine-tuning input for SmolVLM. Finally, we conducted comparative experiments against baseline models and training configurations, assessing performance using both text-generation and classification metrics. This pipeline enabled us to evaluate the model’s ability to provide reliable diagnostic insights and accurately respond to clinical queries, demonstrating the feasibility of small VLMs in specialized medical domains.

## 2. Methods

We adopt SmolVLM-Base as the backbone and fine-tune nine model variants on a dermatology dataset focused on bacterial skin disease, exploring diverse supervision strategies and data scales. The dataset used in this study consists of 2541 bacterial skin disease images, enriched with structured metadata, descriptive captions, and 7623 synthetic QA pairs.

### 2.1. Data Curation

**Figure 1:**
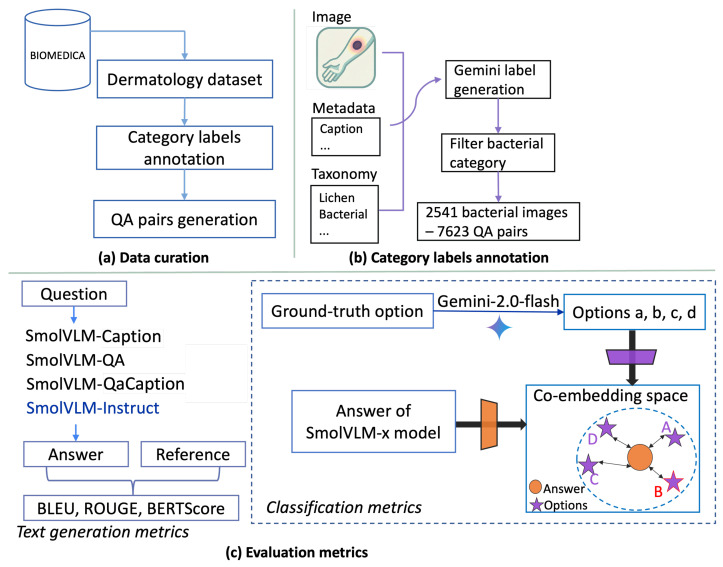
Overview of the data curation workflow, including dataset filtering, category labeling, and QA pairs generation.

The data curation workflow for our PMC-derma-VQA-bacteria dataset is shown in Figure 1a. It consists of three main steps: (1) domain-specific dataset preparation, (2) category label annotation, and (3) QA pair generation. This pipeline extracts a targeted dermatology subset from BIOMEDICA as the initial step for model analysis and enriches it with clinically relevant QA pairs, yielding a domain-specific dataset suitable for fine-tuning.

#### 2.1.1. Domain-Specific Dataset Preparation

We used the BIOMEDICA dataset [24] as the primary source of dermatology images and text. It contains over 24 million image-caption pairs and 30 million image-reference pairs, each enriched with structured metadata and organized under a hierarchical ontology of biomedical concepts.

Using this dataset, we constructed a dermatology-focused subset by selecting images annotated with the primary and secondary concept labels *Clinical Imaging* and *Skin Lesion*. For each selected sample, we retained essential metadata fields including image captions, inline mentions, image dimensions, article license, article title, citation details, and journal source.

#### 2.1.2. Category Labels Annotation

To identify and extract images related to bacterial skin diseases from the dataset, we first defined a taxonomy comprising 29 dermatological categories (Appendix C). This taxonomy served as the target label space for our classification task.

**Figure A1:**
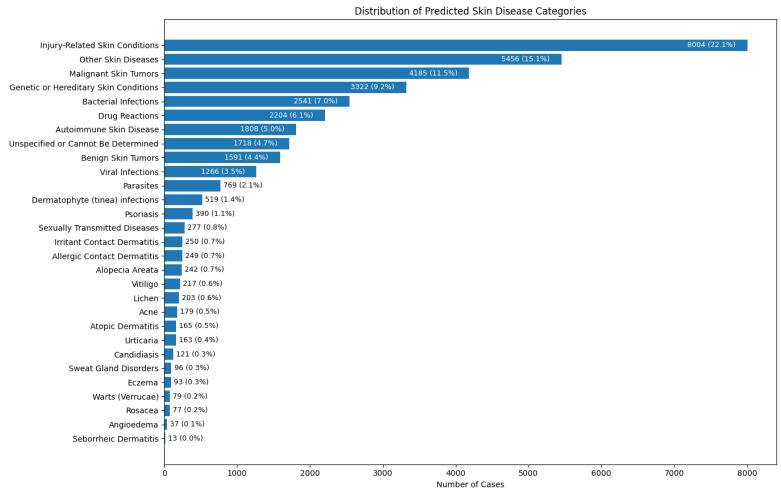
Distribution of predicted skin disease categories in the dataset.

For label annotation, we employed gemini-2.0-flash-exp (accessed between 5 February 2025 and 1 May 2025), supplying it with both the category list and the structured image metadata (Appendix A—Label Generation Prompt). The model was prompted to assign the most appropriate category label to each image, along with a brief explanation, enabling both interpretability and post hoc validation. The distribution of skin disease categories across our dataset is illustrated in Figure A1. As the final step, we filtered the dataset to retain only images classified under bacterial skin disease categories for subsequent analysis, which contains 2541 bacterial disease images and the corresponding 7623 QA pairs.

#### 2.1.3. QA Pairs Generation

Instruction-following has emerged as an effective paradigm for leveraging large language models (LLMs) to generate synthetic data and accelerate downstream model training [25]. We augment each image with three diverse and clinically relevant QA pairs.

To guide the LLM in generating informative and diverse QA pairs, we adopt a prompt-driven instruction-following strategy inspired by DermaSynth [20]. Building upon their prompt template for question generation, we adapt it with our image-level metadata and query GPT-o1 to produce question candidates. The generated questions broadly fall into two categories:

1. General, knowledge-based questions that are independent of specific metadata (Appendix B).
2. Dataset-specific questions that are grounded in the image’s metadata context (Appendix B).

These prompts elicit a variety of question types, including descriptive, diagnostic, comparative, and patient-oriented queries (e.g., “What are the visual features of this lesion?” or “What diagnosis is most likely?”).

For each image, three questions are generated independently, with coverage across both categories to maximize topical diversity. These questions are passed individually to the LLM, ensuring no dependency between answers. The Gemini 2.0 API (model: gemini-2.0-flash-exp, accessed between 18 February 2025 and 1 May 2025) is used to generate responses to each prompt, yielding outputs that reflect dermatological best practices in terminology, structure, and tone.

After synthetic generation, we apply lightweight post-processing to filter out unsuitable or nonsensical QA pairs, such as malformed outputs or answers that are clearly inconsistent with the question or available metadata. We also remove duplicate or near-duplicate QA pairs to improve diversity. This step helps maintain clinical relevance and a coherent linguistic style across the dataset.

The resulting dataset, which we name *PMC-derma-VQA-bacteria*, comprises 2541 images and 7623 QA pairs, each paired with its corresponding caption. Given the limited dataset size, we adopted a 90:10 train–test split to maximize the number of training samples while maintaining an adequate test set for reliable evaluation. The split was performed on a paper-based basis, meaning that all images originating from the same source paper were assigned to the same subset. This strategy prevents potential data leakage across the train and test sets. The final partition yielded 255 images and 765 QA pairs for testing, with the remainder used for training.

### 2.2. Model Fine-Tuning

The primary objective of our fine-tuning process is to enhance the model’s ability to understand and generate domain-specific text by optimizing it to predict the next word in a sequence given the preceding context. Considering the specialized nature of the bacterial skin disease domain, accurate language generation capabilities are essential for effective performance. To run the training, we used Google Colab with an NVIDIA A100 GPU with 40 GB of VRAM. This setup already provided the necessary computational resources to fine-tune the SmolVLM-Base model with parameter 2 B. The SmolVLM family comprises lightweight multimodal models spanning 1B–7B parameters, designed to deliver competitive vision language performance with reduced computational and memory cost. Within this family, the 2B variant is renowned for its efficient memory footprint, making it a suitable choice for fine-tuning under our resource constraints.

To facilitate the adaptation of the SmolVLM-Base model to the domain of bacterial skin disease and enhance its proficiency to generate contextually appropriate answers from visual inputs, Low-Rank Adaption (LoRA) was implemented for parameter-efficient fine-tuning [26]. This methodology is well-suited for resource-constrained settings, as it effectively reduces the number of trainable parameters while maintaining the pre-existing knowledge encapsulated within the base model [27]. In this project, low-rank adapters were integrated into the q_proj and v_proj layers of the model architecture. These adapters were configured with a rank of 8, a scaling factor of α=32, and a dropout rate of 0.1. These hyperparameters were selected based on prior research and a set of small-scale pilot experiments conducted at the beginning of this study to identify suitable configurations.

The adapters were trained with causal language modeling (CLM) objectives, while the base model parameters were kept frozen. The training schedule consisted of 2 epochs, utilizing a per-device batch size of 1 and gradient accumulation over 4 steps, resulting in an effective batch size of 4.

As shown in Figure 1c, to evaluate the effectiveness of different supervision strategies in low-resource fine-tuning, we trained three variants of the model: (1) using only synthetic QA pairs (QApairs); (2) using only figure image captions (Caption); and (3) combining synthetic QA pairs and captions (QaCaption). For each supervision type, the training set was further stratified into three sizes—500, 1000, and the full 2286 training samples—to examine the impact of data size on model performance. This setup not only enables a comparison between structured QA pairs, captions, and their combination, but also allows us to assess whether small-parameter VLMs can maintain strong performance with limited training data.

### 2.3. Evaluation Benchmarks

Building on the 10% held-out test split, we employed a comprehensive evaluation framework that combines text-generation and classification metrics to assess the performance of our fine-tuned models. For comparison, we used SmolVLM-Instruct [28], a model trained on general-purpose data, as the baseline.

#### 2.3.1. Text Generation Metrics

For the evaluation of text generation, we employed a combination of widely recognized metrics, including BLEU [29], ROUGE [30], and BERTScore [31]. BLEU calculates lexical precision by measuring the overlap of n-grams between candidate and reference texts. In contrast, ROUGE adopts a recall-oriented approach: ROUGE-L leverages the longest common subsequence (LCS) to assess structural similarity, while ROUGE-N directly quantifies the proportion of n-grams from the reference that are present in the candidate.

Despite their effectiveness, both BLEU and ROUGE operate at the surface level of n-gram matching, which may overlook semantically equivalent paraphrases. To overcome this limitation, we incorporated BERTScore, which uses contextual embedding from the BERT model to compute token-level semantic similarity.

Together, these metrics provide a comprehensive evaluation framework: BLEU rewards lexical precision, ROUGE emphasizes content recall, and BERTScore captures semantic similarity. Such a multifaceted evaluation is crucial for LLMs in the medical domain, where outputs must not only be lexically accurate and complete, but also semantically aligned with clinical standards [32,33].

#### 2.3.2. Classification Metrics

For classification tasks, we designed a structured evaluation framework that transforms free-form generation into a semantic matching task. Instead of directly prompting the model to select from predefined options, we employed a two-step process to assess its ability to generate contextually relevant responses and align them with plausible choices. Here, the plausible choices refer to a single correct answer accompanied by several semantically relevant but incorrect distractors, forming a single-choice QA setting. This approach provides a more reliable measure of contextual understanding and response accuracy. Prior work shows that answer matching achieves higher agreement with human judgments than multiple-choice scoring and remains effective for smaller models [34]. As illustrated in Figure 1c, the pipeline proceeds as follows:

1. Distractor generation: Using the gemini-2.0-flash-exp model, we generated 3 semantically relevant distractor options for each ground-truth answer in the test dataset. These distractors were designed to mimic common misdiagnoses or plausible but incorrect clinical interpretations. For instance, confusing folliculitis with acne vulgaris in the context of bacterial skin diseases.
2. Free-form response generation: We prompted the fine-tuned model to generate an open-ended response based on the input image and question, aiming to demonstrate its ability to synthesize information without being explicitly constrained to predefined answer choices.
3. Semantic matching: A semantic encoder was used to compute the semantic similarity score between the generated response and the four candidate options. The option with the highest score was used as the model’s prediction, and classification accuracy was calculated as the proportion of correct predictions.

## 3. Results

In this study, we trained three model variants with different supervision signals across three training-set sizes, yielding nine fine-tuned models. We evaluate their performance on bacterial disease images using both text-generation and classification metrics under identical experimental settings, with detailed results summarized in Table 1. Overall, Table 1 shows three conclusions: (i) QA-only supervision achieves the best text-generation quality (highest BLEU/ROUGE and BERTScore); (ii) combining QA pairs with captions yields the highest classification accuracy (70.20%); and (iii) performance generally improves as the training set increases across supervision settings.

For QApairs-only models, performance improved steadily with dataset size, achieving an accuracy of 61.96%, 66.41%, and 68.63% on datasets of sizes 500, 1000, and 2286, respectively. The corresponding BERTScore reached up to 90.19.

Combining both captions and QA pairs (QaCaption models) resulted in the strongest improvements in accuracy compared to their individual counterparts, with QaCaption-2286 achieving the highest accuracy of 70.20%. In contrast, the weakest performance was observed on the smallest datasets, Caption-500 and QApairs-500 (both 61.96%), highlighting the benefit of combining supervision signals, especially as data scale. In terms of BERTScore, QaCaption models performed slightly better than Caption-only models but still lower than QApairs-only models. QApairs-only models, however, clearly outperformed the others in BLEU and ROUGE metrics.

**Figure 2:**
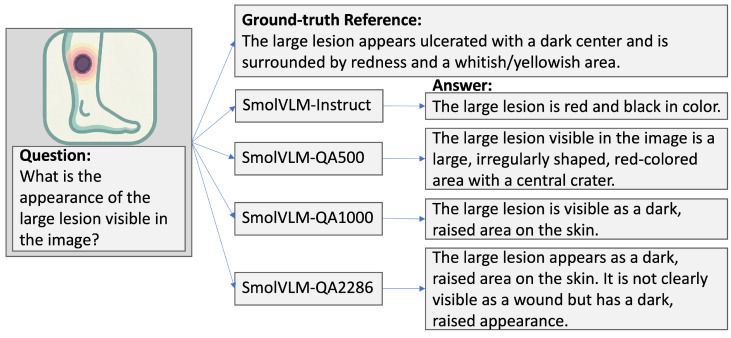
Illustrative example showing the question, ground-truth reference, and outputs from the baseline SmolVLM-Instruct model and three fine-tuned models trained on datasets of increasing size. The example highlights that, in line with the quantitative results in Table 1, supervision type has a more pronounced impact on semantic alignment and clinical relevance than dataset size. An icon is shown instead of the actual image.

In contrast, the baseline SmolVLM-Instruct model already achieved a relatively high accuracy of 68.24%, which is close to that of the fine-tuned models. However, it showed a significant drop in BLEU and ROUGE scores while maintaining competitive performance on BERT Score (87.69%). This competitiveness, however, should be interpreted cautiously since BERTScore values across all models lie within a narrow range (87.5–90.2%), indicating limited discriminative sensitivity of this metric in our setting. Figure 2 illustrates an example comparison between the SmolVLM-Instruct and fine-tuned models, showing how the latter generates answers more aligned with the detailed ground-truth reference in clinical image interpretation.

## 4. Discussion

Previous studies have demonstrated the feasibility of domain-adapted VLMs for generating clinically relevant narratives in dermatology [13,35,36]. Building on this, Lozano et al. [37] showed that fully fine-tuning the small-scale SmolVLM with multiple VQA datasets and a subset of the BIOMEDICA dataset can yield performance surpassing many smaller models and approaching that of LLaVA-Med [38], a 7B-parameter model. In our study, we trained nine models under different supervision types and dataset sizes, and found that even with constrained data resources, appropriately aligned small-scale VLMs can produce semantically accurate and clinically meaningful descriptions, outperforming general-domain models in clinical narrative generation. BERTScores in Table 1 show minimal variation across different training set sizes, but exhibit more noticeable changes across different supervision types. This indicates that dataset size has a limited effect on semantic alignment quality, whereas supervision modality plays a more decisive role. Consistent with previous studies [28,39], this suggests that small-parameter VLMs are well-suited for low-resource settings with limited fine-tuning data.

**Figure 3:**
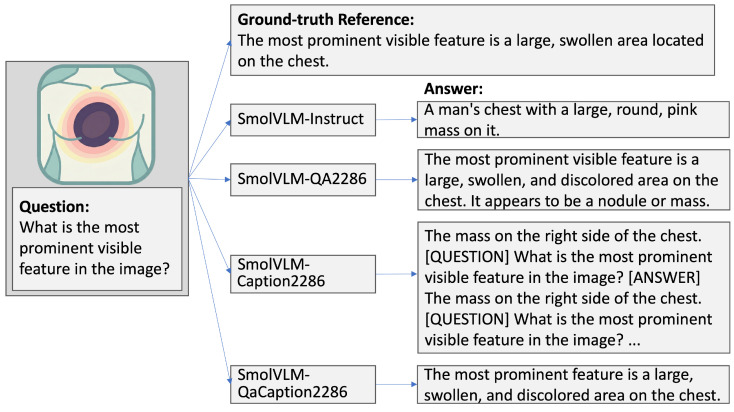
Illustration of model responses trained on different dataset types with equal sample sizes. Verbose, descriptively rich answers (SmolVLM-QA2286) tend to achieve higher BLEU scores due to greater lexical overlap, whereas concise, label-aligned outputs (SmolVLM-QaCaption2286) yield higher accuracy under strict semantic matching. SmolVLM-Caption2286 occasionally produces repetitive patterns. An icon is shown instead of the actual image.

Regarding the impact of supervision strategies on small-scale VLMs for dermatology, the results in Table 1 reveal that high ROUGE or BLEU scores do not necessarily translate to high accuracy. For example, the QApairs-based model trained on the full dataset achieved the highest ROUGE-1 (0.4060) and ROUGE-L (0.3417) scores, yet its accuracy was moderate (0.6863). QaCaption achieved the highest accuracy (0.7020) despite lower ROUGE scores, and the instruct model ranked second in accuracy (0.6824). This discrepancy arises because ROUGE and BLEU reward lexical overlap, favoring verbose or descriptive outputs, whereas accuracy here measures exact semantic matching to a single correct option. QaCaption models, exposed to richer and more semantically focused training data, tend to produce concise, canonical outputs aligned with the label space, boosting accuracy under strict matching even without maximizing lexical similarity. A plausible mechanism is that combining captions with QA pairs implicitly regularizes the output style and vocabulary: captions encourage standardized clinical terminology and canonical naming, while QA pairs reinforce discriminative, label-oriented responses. This synergy benefits closed-set classification under strict matching, but it can reduce descriptive richness and lexical diversity in free-text answers, thereby limiting improvements in BLEU/ROUGE compared to QA-only supervision. For instance, in Figure 3, both the instruct and QaCaption-2286 models produced concise outputs that aligned well with ground-truth labels, underscoring the practical advantage of brevity in semantic matching. In contrast, QA-only supervision aligns with the question-conditioned generation format used during evaluation, effectively functioning as an instruction-tuning signal that encourages focused, canonical responses and therefore yields higher ROUGE and BLEU scores.

Furthermore, context length and model scale are critical considerations for clinical deployment. In clinical practice, overly verbose responses are less useful because healthcare providers prioritize concise, accurate descriptions over lengthy narratives. This underscores the practicality of small-scale VLMs. Despite VLMs’ limited parameter size, they can produce comprehensive yet focused responses, making them well-suited to time-critical, resource-constrained medical settings.

This study has limitations. Our evaluation is research-based and conducted on a curated PMC-OA figure dataset rather than within a real clinical workflow. Therefore, the reported results may not fully reflect deployment conditions such as smartphone photographs, diverse skin tones, varied acquisition settings, and clinician interaction. A more realistic clinical validation should be conducted prospectively with dermatologist oversight, including external validation on real-world clinical data, comparison against clinician baselines, and safety checks for hallucinations and inappropriate recommendations.

We also note that synthetic QA pairs are generated by LLMs (Gemini), which may introduce biases in question formulation, answer phrasing, and vocabulary normalization. Such stylistic regularities can influence n-gram overlap–based generation metrics, such as BLEU and ROUGE, and may limit generalization to the more diverse language observed in real clinical interactions. Incorporating clinician-authored queries and evaluating on externally collected, human-written QA are important directions for future work. While caption supervision can enhance the richness and visual grounding of generated language, it may also introduce noise. In certain cases, the model reuses irrelevant caption fragments, such as photographer credits, indicating difficulty distinguishing clinically meaningful visual cues from textual artifacts. This highlights the need for improved caption integration strategies, such as modular supervision heads or input masking, to better separate visual grounding from format contamination.

Another notable limitation concerns the tendency of models to produce repetitive outputs. As illustrated in Figure 3, the fine-tuned models occasionally repeated phrases or partially mirrored the input prompt. This observation is consistent with prior reports that smaller models are more susceptible to repetition, whereas increasing model scale has been shown to mitigate such effects [40]. Accordingly, future work may investigate the use of moderately larger architectures, such as models with 10–20 billion parameters, as a potential balance between improved performance and computational feasibility. These would still be relatively small compared to trillion-parameter models.

## 5. Conclusions

In conclusion, this study demonstrates the substantial potential of agentic small-scale VLMs for clinical decision support in dermatology, particularly in subfields focusing on bacterial skin disease, where sample sizes are obvious. Using 2286 bacterial skin disease images and 6858 corresponding QA pairs, we fine-tuned the SmolVLM-Instruct model with different supervision strategies. The fine-tuned models with synthetic data significantly outperformed the base SmolVLM-Instruct model across our evaluation metrics. Moreover, by comparing the impact of different data sizes, we observed that some of our fine-tuned models achieved comparable or even superior performance when fine-tuned with a small dataset of 500 images, compared with fine-tuning on the full dataset. Although the highest accuracy reached was 70.2%, it consistently increased with the richness of the training corpus. These findings suggest that future work could maintain the same dataset scale while incorporating instructional-style data to enrich the diversity of the training data.

Our study lays the foundation for developing a dermatology-focused AI assistant that can be embedded in a multi-agent medical system under low-resource constraints. This system could extend beyond bacterial skin diseases and be evaluated on broader real-world dermatology tasks, including lesion recognition, morphology description, and differential diagnosis. More broadly, the same pipeline may generalize to other medical imaging specialties where labeled data are limited and lightweight, explainable assistants are needed.

## Figures and Tables

**Table 1:** Performance comparison of models against ground-truth annotations on bacterial disease images. Bold values indicate the best performance in each metric.

Model-Data	Accuracy	BLEU	ROUGE-1	ROUGE-L	BERT Score
QApairs-500	61.96%	**11.46**%	**40.78**%	**35.12**%	**90.19**%
QApairs-1000	66.41%	10.52%	39.30%	33.28%	89.86%
QApairs-2286	68.63%	10.94%	40.60%	34.17%	90.17%
Caption-500	61.96%	5.23%	29.13%	24.24%	87.58%
Caption-1000	63.40%	5.60%	30.65%	25.05%	87.68%
Caption-2286	65.36%	6.18%	31.69%	26.08%	87.64%
QaCaption-500	64.71%	8.20%	34.71%	29.32%	88.74%
QaCaption-1000	67.45%	7.69%	34.16%	28.54%	88.75%
QaCaption-2286	**70.20**%	7.87%	34.45%	28.56%	88.79%
SmolVLM-Instruct	68.24%	3.59%	22.93%	19.10%	87.69%

## Data Availability

The original data used in the study are openly available on Hugging Face at https://huggingface.co/BIOMEDICA (accessed on 1 February 2025).

## Abbreviations

The following abbreviations are used in this manuscript:

LLM	large language model
VLM	vision language model
SOTA	state-of-the-art
PMC-OA	PubMed Central Open Access
QA	question–answer
AI	artificial intelligence
CLM	causal language modelling
LoRA	low-rank adaptation
VQA	visual question–answer
QApairs	dataset of synthetic question answer pairs generated
Caption	dataset of image captions from the BIOMEDICA dataset
QaCaption	dataset of synthetic QA pairs and image captions

## Appendix A. Prompts

### Appendix A.1. Distractor Option Generation Prompt

To generate distractor options, we provided Gemini with the original question and its ground-truth answer using the following prompt:

***** INSTRUCTIONS ***** You are a medical assistant. Given the following question and its correct answer, generate 3 plausible but incorrect options. **** CONTEXT ***** Question: {question} Correct Answer: {correct_answer} **** RESPONSE FORMAT ***** - Option 1: … - Option 2: … - Option 3: …

### Appendix A.2. Label Generation Prompt

During label generation, we use the following instruction prompt to annotate the corresponding label to each image:

***** INSTRUCTIONS ***** You are a dermatology expert. For each of the following image metadata entries, determine the most appropriate skin disease category from the list below. Provide your prediction along with a concise and well-reasoned explanation. Skin Disease Categories: {label_block} **** CONTEXT ***** {metadata_context} **** QUESTION ***** Please determine which of the following skin disease categories this image most likely belongs to. **** RESPONSE FORMAT ***** Predicted Category (Number + Name): <Number>. <Name> Brief Reasoning: <Explanation>

## Appendix B. Example Questions

All questions are available in the code repositories.

### Appendix B.1. Example General Questions

- What are the most prominent visual features in this lesion/disease? - Based on the image, what might be the diagnosis? - What conditions might present similarly to the lesion/disease in this image? - How would you rank the most likely alternative diagnoses for this lesion/disease? - How would you explain the lesion/disease in this image to a worried patient? - What language would you use to describe this lesion/disease in a patient consultation? - What follow-up procedures might be necessary for this lesion/disease? - What further evaluations are essential for this lesion/disease’s treatment or diagnosis?

### Appendix B.2. Example Dataset Specific Questions

- Based on the appearance of this lesion/skin disease, what condition might it represent? - Considering its morphology, what might this lesion/disease indicate? - What does the lesion / disease’s distribution of pigmentation suggest? - Could this lesion/skin disease represent a malignant condition? Why or why not? - What dermatological disorders might resemble the lesion/skin disease in this image?

## Appendix C. Skin Disease Categories

### List of 29 Dermatological Categories Used for Classification

1. Acne 2. Rosacea 3. Seborrheic Dermatitis 4. Parasytes 5. Irritant Contact Dermatitis 6. Allergic Contact Dermatitis 7. Urticaria 8. Angioedema 9. Drug Reactions 10. Warts (Verrucae) 11. Dermatophyte (tinea) infections 12. Candidiasis 13. Psoriasis 14. Lichen 15. Alopecia Areata 16. Vitiligo 17. Atopic Dermatitis 18. Eczema 19. Benign Skin Tumors 20. Malignant Skin Tumors 21. Bacterial Infections 22. Viral Infections 23. Sexually Transmitted Diseases 24. Autoimmune Skin Disease 25. Genetic or Hereditary Skin Conditions 26. Sweat Gland Disorders 27. Injury-Related Skin Conditions 28. Other Skin Diseases 29. Unspecified or Cannot Be Determined
